# Maternal Prepregancy BMI and Lipid Profile during Early Pregnancy Are Independently Associated with Offspring's Body Composition at Age 5–6 Years: The ABCD Study

**DOI:** 10.1371/journal.pone.0094594

**Published:** 2014-04-16

**Authors:** Maaike G. J. Gademan, Marloes Vermeulen, Adriëtte J. J. M. Oostvogels, Tessa J. Roseboom, Tommy L. S. Visscher, Manon van Eijsden, Marcel T. B. Twickler, Tanja G. M. Vrijkotte

**Affiliations:** 1 Department of Public Health, Academic Medical Center - University of Amsterdam, Amsterdam, The Netherlands; 2 Department of Obstetrics and Gynaecology, Academic Medical Center, Amsterdam, The Netherlands; 3 Department of Clinical Epidemiology, Biostatistics and Bioinformatics, Academic Medical Center, Amsterdam, The Netherlands; 4 Research center for the prevention of overweight, Windesheim and VU University, EMGO+, Zwolle/Amsterdam, The Netherlands; 5 Department of Health Sciences, VU University, Amsterdam, The Netherlands; 6 Department of Epidemiology, Documentation and Health Promotion, Public Health Service of Amsterdam (GGD), Amsterdam, the Netherlands; 7 Department Endocrinology, Diabetology and Metabolic Diseases, Antwerp University Hospital, Antwerp, Belgium; INRA, France

## Abstract

**Background:**

There is growing evidence that disturbances in maternal metabolism and, subsequently, intrauterine conditions affect foetal metabolism. Whether this has metabolic consequences in offspring later in life is not fully elucidated. We investigated whether maternal pre-pregnancy body mass index (pBMI) is associated with offspring's adiposity at age 5–6 years and whether this association is mediated by the mother's lipid profile during early pregnancy.

**Methods:**

Data were derived from a multi-ethnic birth cohort, the Amsterdam Born Children and their Development (ABCD) study (inclusion 2003–2004). During early gestation mothers completed a questionnaire during pregnancy (pBMI) and random non-fasting blood samples were analysed for total cholesterol (TC), triglycerides (TG), apolipoprotein A1 (ApoA1), apolipoprotein B (ApoB) and total free fatty acids (FFA) in early gestation. At age 5–6 years, child's BMI, waist-to-height-ratio (WHtR) and fat% were assessed.

**Results:**

Only non-diabetic mothers with at term-born children were included(n = 1727). Of all women, 15.1% were overweight(BMI: 25–29.9 kg/m2) and 4.3% were obese(BMI≥30 kg/m2). After adjustments for confounders, every unit increase in pBMI was linearly associated with various offspring variables: BMI(β 0.10; 95% CI 0.08–0.12), WHtR*100(β 0.13; 95% CI 0.09–0.17), fat%(β 0.21; 95% CI 0.13–0.29) and increased risk for overweight(OR:1.15; 95% CI 1.10–1.20). No convincing proof for mediation by maternal lipid profile during early gestation was found. Moreover, maternal FFA was associated with the child's fat percentage, BMI and risk for overweight. Maternal ApoB and TC were positively associated with the offspring's fat percentage and maternal TG was positively associated with their children's WHtR.

**Conclusions:**

Both pBMI and maternal lipids during early pregnancy are independently related to offspring adiposity.

## Introduction

Currently, 10% of the world's school-aged children are obese or overweight and this percentage is progressively growing [1], [2]. Long-term health consequences of childhood obesity are adult obesity and, in adulthood, its related morbidities including premature death, cardiovascular disease and depression [3], [4] Hence, understanding the origin of childhood obesity is important for prevention in adulthood. Lifestyle, such as physical activity, is an acknowledged determinant, but foetal metabolic priming may also be a contributing factor.

Overweight or obese mothers have a higher risk of giving birth to children with a higher body mass index (BMI) [5], [6]. The Developmental Origin of Adult Diseases (DOHaD) hypothesis propose that maternal metabolic perturbations, and its subsequent intrauterine conditions, can program the fetus to be more prone for obesity [7]–[9]. However, the exact biological mechanisms in humans need to be more explored (as evidence for the existence of such a foetal metabolic programming mainly emerged from animal studies).

A more pronounced maternal lipid profile is more often observed in obese women [10]. During pregnancy this obese phenotype is even more exaggerated giving metabolic priming of the foetus an opportunity; programming of offspring obesity by maternal obesity [9], [11]–[16]. Rodents, fed a cafeteria diet, had an exaggerated lipid profile and this intrauterine conditions negatively affected offspring's neuroendocrine system resulting in hyperphagia, altered adipocyte function, accelerated weight gain after weaning, and adiposity [9], [17]. In humans, observations from our group and others [12], [16], [18] show that high maternal triglyceride (TG) levels increase the risk of being large for gestational age and a low maternal TG levels increase the risk for being small for gestation [11], both are known to be associated with childhood obesity.

Against this background, we evaluated in the ABCD cohort whether the maternal lipid profile in early pregnancy is associated with maternal pre-pregnancy BMI (pBMI) and the occurrence of adiposity in the offspring at age 5–6 years.

## Materials/subjects and Methods

### Study population

This study is part of the ABCD cohort (Amsterdam Born Children and their Development), a large community based birth cohort [14]. Approval of the study was obtained from the Central Committee on Research Involving Human Subjects in the Netherlands, the medical ethics review committees of the Academic Medical Center, Amsterdam, the VU University Medical Center Amsterdam and the Registration Committee of the Municipality of Amsterdam. All women provided written informed consent. Parents or caretakers provided written informed consent for the health check of the 5–6 year old children in the questionnaire that was send around two weeks after the child's fifth birthday. This questionnaire was only send to mothers who initially gave permission for follow-up. This informed consent procedure was approved by the committees of the Academic Medical Center, Amsterdam, the VU University Medical Center Amsterdam and the Registration Committee of the Municipality of Amsterdam.

Between January 2003-March 2004 all pregnant women living in Amsterdam were invited to participate in the ABCD study at the first visit to their obstetric care provider. Women filled out a questionnaire containing questions on sociodemographic characteristics, medical history, lifestyle and dietary habits (16 weeks of gestation; IQR 12–20 weeks). All women who agreed to complete the questionnaire (n = 8266) were asked to attend the ABCD biomarker study. For this latter study, an extra blood sample (10 ml EDTA and 9 ml serum) was taken during the first prenatal visit(13 weeks of gestation; IQR, 12–14 weeks)(n = 4389).

Birth outcomes (gestational age at birth, sex, birth weight and congenital abnormalities) were obtained through Youth Health Care (YHC). After the child's 5th birthday, a questionnaire was sent to the parents who gave permission for follow-up (n = 3501). After that a health check was done at the primary school of the child (age 5.7 years ±0.5 SD)(n = 1956).

**Figure 1:**
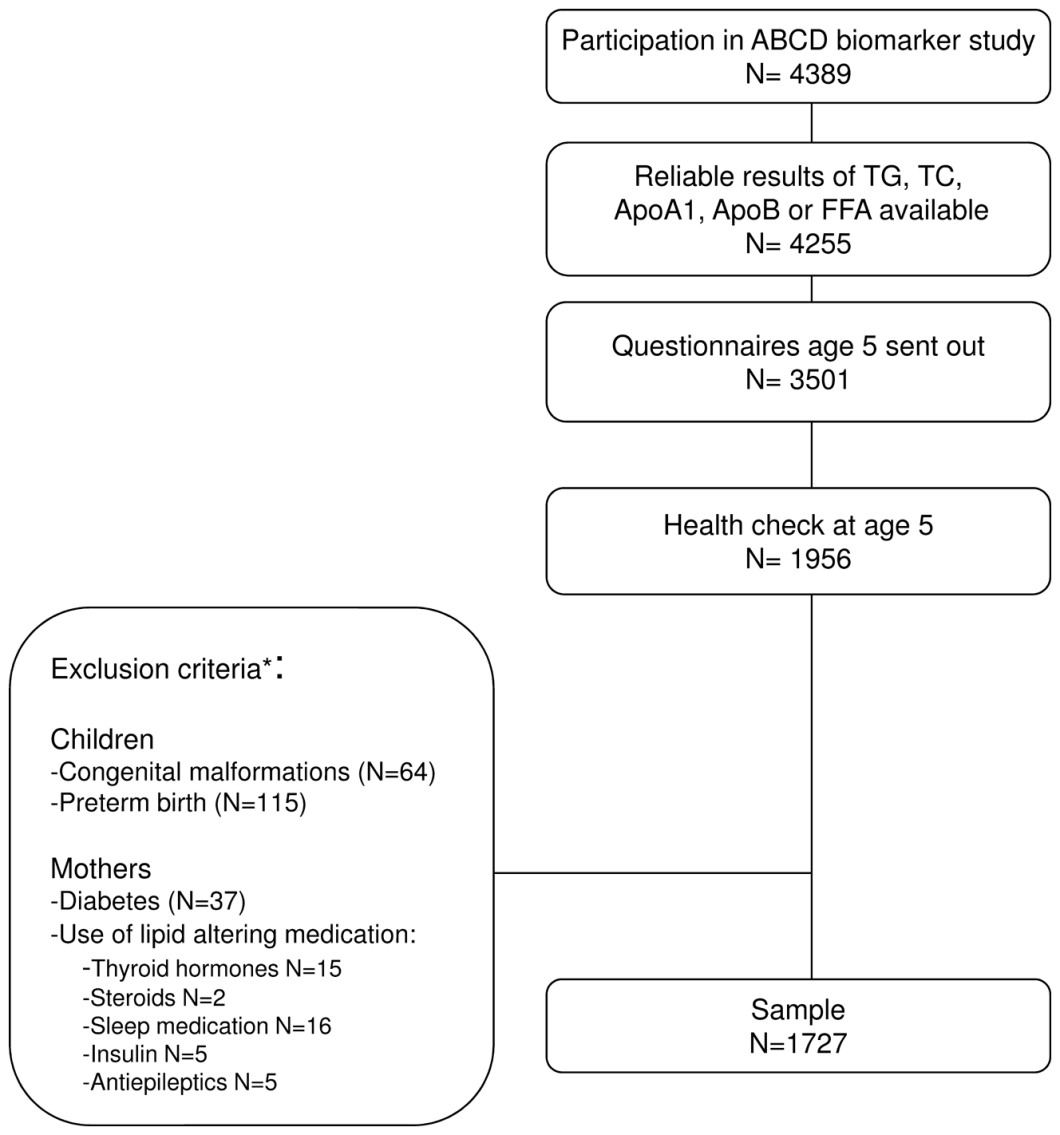
Flow chart of the study population. *Numbers sum to more than 229 as more than one exclusion criterion per mother was sometimes present.

Women who gave birth to twins, delivered preterm (<37 weeks of gestation), with diabetes or used lipid-altering medication (e.g. thyroid hormones, steroids, sleep medication, insulin use and antiepileptic drugs) were excluded(n = 229)(Figure 1). Children with congenital malformations were also excluded. Finally, 1727 mother-child pairs were included (Figure 1).

### Maternal lipid measurements and pBMI

Nonfasting blood sampling was conducted. For each woman a blood sample was taken in a 9-mL Vacuette (Greiner BV, Alphen aan de Rijn, the Netherlands) for the preparation of serum. From this sample triglycerides TG, total cholesterol (TC), apolipoprotein A1 (ApoA1), apolipoprotein B (ApoB) and free fatty acids (FFA) were determined as follows. TG was tested with the glycerol-3-phospate oxidase PAP method. TC was assessed using the cholesterol oxidase PAP method on a Hitachi 912 analyser (Roche Diagnostics, Mannheim, Germany). The interassay coefficient of variation was 2.3% for TG and 1.0% for TC. ApoA1, ApoB and FFA analyses were done with an Abbot Architect CI 8200 (Abbott Laboratories, Limited. Saint-Laurent, Québec, Canada). A turbidimetric technique was used for analysing ApoA1 and ApoB. The FFA analyses were done with a combination of enzymatic and spectrophotometry/colorimetry (reagent by Wako Pure Chemical Industries Ltd. (Osaka, Japan)). The interassay coefficient of variation was 1.6% for ApoA1, 1.6% for ApoB and 3.4% for FFA.

pBMI was based on pre-pregnancy height and weight as reported in the pregnancy questionnaire. In the whole ABCD cohort missing values were imputed maternal height (1.8% missing in the current study population) and pre-pregnancy weight (8.2% missing in the current study population) by a random imputation procedure using linear regression analysis and other variables known to be associated with maternal height and weight, respectively [19].

### Child measurements

Trained research assistants performed the health check for the 5-year-old children. The physical measurements included height, weight, and waist circumference. Height was determined to the nearest millimetre using a Leicester portable height measure (Seca, Hamburg, Germany) and weight to the nearest 100 g using a Marsden MS-4102 weighing scale (Oxfordshire, United Kingdom). Waist circumference was measured midway between the costal border and the iliac crest to the nearest millimetre using a Seca measuring tape. Fat mass was measured by arm-to-leg bioelectrical impedance analysis (Bodystat 1500 MDD machine (Bodystat Inc, Douglas, UK)).

### Definitions of measurements

Outcome measures were the anthropometric measurements of the child at age 5–6 years and the lipid profile of the mother during early pregnancy. BMI was calculated as the child's weight in kilograms divided by their height in meters squared. We also computed the waist-to-height ratio (WHtR); during the analysis WHtR was multiplied by 100. Fat percentage was based on calculations from our validation study [20]. Because the ApoB/ApoA1 ratio is a reliable predictor of heart disease [21], we computed this ratio to evaluate its influence on adiposity of the child. BMI of the child was analysed as a dichotomous outcome value (overweight yes/no) defined according to the International Obesity Task Force guidelines [22].

### Covariables

The following covariables related to the mother were taken into account: maternal age(continuous), parity (primiparous yes or no), maternal height (continuous), hypertension (no hypertension, pre-existing or pregnancy induced), ethnicity (country of birth, Western/non-Western), smoking during pregnancy (smoker/non-smoker), alcohol use during pregnancy (drinker/non-drinker) and years of education after primary school (continuous). Maternal characteristics were self-reported in the pregnancy questionnaire, except for hypertensive status which was obtained by combining recordings from the Dutch perinatal registration, medication use and self-reported hypertension [23]. Covariables from the child were sex of the child, duration of exclusive breastfeeding (no breastfeeding, <1 month, 1–3 months, >3 months) screen (computer and television) time hours/day at age 5–6 years (hours, continuous) and saturated fat intake (grams/day, continuous) at age 5–6 years. Duration of breastfeeding was available from the infancy questionnaire received when the child was aged 3 months and from the Child Health Care Registration. This prospectively collected information was combined with retrospective information of the 5-year questionnaire to complete the data (19.9% was from 5-year questionnaire). Number of screen-time hours was obtained from the 5-year questionnaire as well as the child's saturated fat intake. Which was assessed by the food frequency questionnaire, which is validated in children [24]. As both the number of missings in screen time-hours and satured fat intake were relatively high, 7.8% and 15.0% respectively (in the current study population), these values were imputed in the ABCD biomarker study (n = 4389) by a random imputation procedure using linear regression analysis and all variables used in the current study.

Because we considered birth weight and gestational age as intermediates between pBMI, maternal lipid profile and child's adiposity, no adjustment was made for birth weight [5], [6], [11].

### Statistical analysis

Analyses were performed with R version 2.14.1 and SPSS version 16.0 (SPSS Inc, Chicago, Ill, USA). No departure from linearity was observed.

Associations were explored using linear (where applicable on continuous) and logistic (where applicable on dichotomous outcomes) regression models. First, the association between maternal pBMI (continuous measure) and lipids was explored (a), adjusting for gestational age at blood sampling. Next, we explored the relation between maternal lipids and the adiposity of the child (b), adjusting for gestational age at blood sampling, sex and age of the child. Then we explored the association between maternal pBMI and the adiposity of the child, adjusting for sex and age of the child (c). Finally, all analyses (a–c) were repeated (model 2) with adjustment for potential confounders. The confounders were based on previous literature, and were defined a priori. The following potential confounders were identified: age mother, parity, ethnicity, height, years of education, alcohol, smoking, and hypertension and from the child, duration of exclusive breastfeeding, screen time hours/day and saturated fat intake Subsequently, logistic regression analyses were conducted following the same procedure as the linear regression models, to explore the association between pBMI, lipids and the risk of offspring's overweight.

In order to conclude that maternal lipids were intermediates in the association between maternal pBMI and child's adiposity we first conducted a mediation analyses according to the causal-steps analysis [25]. According to the causal-steps analyses all three associations (a–c) should be statistically significant after adjustments, and when analysing the association between pBMI and adiposity of the child (c), the addition of the maternal lipid variables to the full adjusted models (Model 2) should attenuate the association into a statistically non-significant association between pBMI and child's adiposity [25].

**Figure 2:**
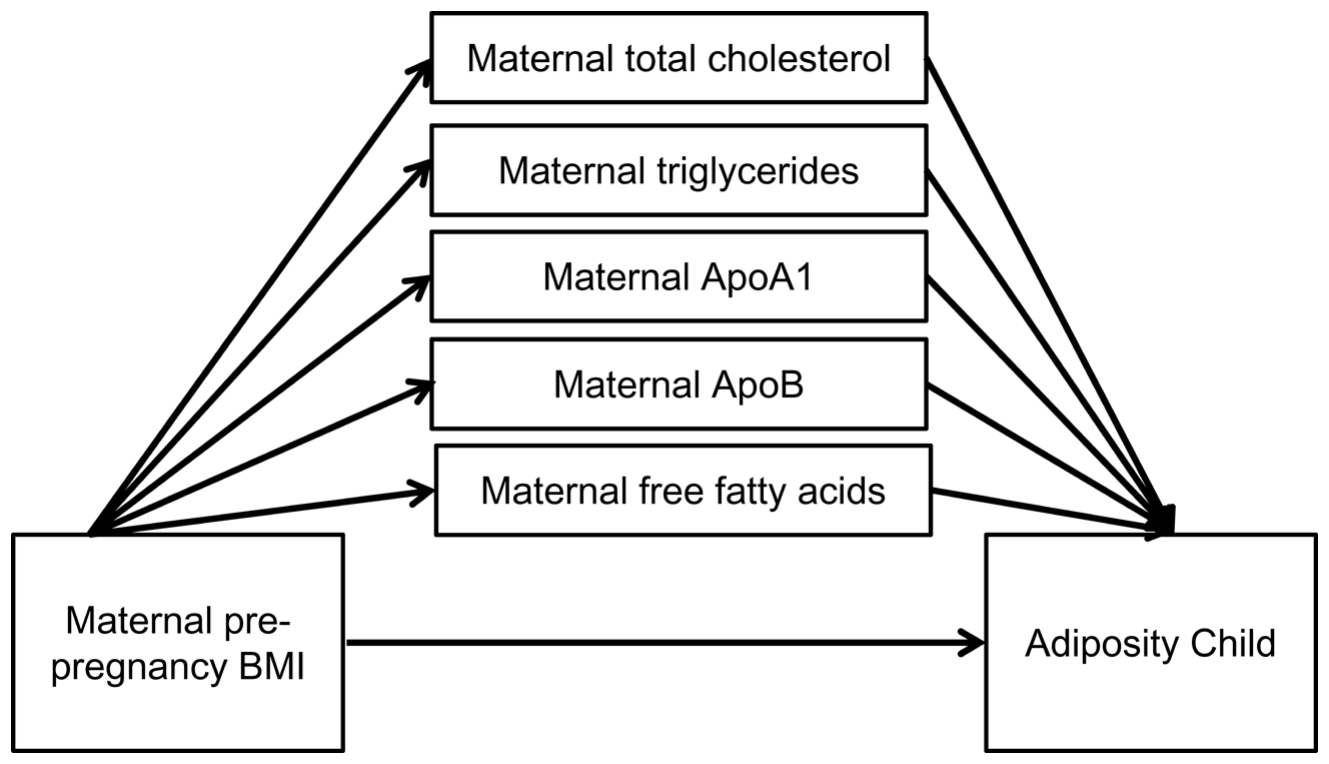
Indirect effect of maternal pre-pregnancy BMI on adiposity of the child through the maternal lipid profile.

In addition we also assessed whether maternal lipids were mediators between maternal pBMI and child's adiposity by testing the significance of the indirect or mediated effect computed as the product of regression coefficient estimates using a bootstrapping approach with a multiple mediator model (Figure 2) [26], [27]. All the maternal lipid variables were included in this model. The resampling rate was set at 10.000.

*P*-values <0.05 were considered statistically significant. Medians are presented with IQRs and means with SD.

## Results

### Study population

The present study consisted of 1727 mother-child pairs (Figure 1). Compared with the women lost to follow-up the included women were older, more often of Dutch origin, had a higher education level, and breastfed their children for longer (Table S1).

Of all women, 15.1% were overweight and 4.3% were obese (Table 1). Overweight/obese women were more likely to have hypertension, had fewer years of education after primary school, were less often primiparous, and less often consumed alcohol. Their lipid profile (with the exception of ApoA1 and TC) was higher than that of women with a normal weight. Furthermore, obese women were less likely to breastfeed their babies. Of all children, 8.3% were overweight or obese, mean BMI, WHtR and fat percentage were 15.4±1.4 kg/m^2^, 0.45±0.03 and 23.6±6.2%, respectively. At age 5–6 years, children from overweight/obese mothers were more likely to have a higher BMI, WHtR and fat percentage compared with children from mothers of the normal pBMI group (Table 1). Also children of overweight/obese mothers had a higher screen time than children from mothers of the normal pBMI group.

**Table 1:** Maternal and child characteristics by maternal pBMI (n = 1727).

	pBMI<18.5	pBMI 18.5–24.9	pBMI 25–29.9	pBMI≥30
	n = 66	n = 1326	n = 260	n = 75
	3.8%	76.8%	15.1%	4.3%
*Maternal characteristics*	SD	SD	SD	SD
Age (years) n = 1727	30.8 (3.9)	32.1 (4.2)	31.2 (5.1)	31.5 (4.6)
Ethnicity (%) n = 1727				
Dutch	65.2	81.4	66.5	56.0
Turkish	1.5	2.1	2.3	1.3
Moroccan	3.0	2.2	11.5	12.0
Other non-Western	18.2	7.5	14.6	26.7
Other Western	12.1	6.9	5.0	4.0
Primiparous (% yes) n = 1727	59.1	58.8	50.0	40.0
Education after primary school (years) n = 1720	9.6 (4.4)	10.4 (3.3)	9.0 (3.8)	7.7 (3.9)
Height (cm) n = 1727	168.3 (8.2)	170.3 (6.8)	168.2 (7.2)	166.0 (7.0)
Hypertension (%) n = 1727				
Pre-existing	0	1.7	5.8	5.3
Pregnancy-induced	1.5	7.6	13.5	13.3
Smoking (yes, %) n = 1727	4.5	8.9	6.2	10.7
Alcohol (yes, %) n = 1726	31.8	33.3	16.5	16.0
***Lipids***				
Total cholesterol (mmol/L) n = 1673	4.88 (0.71)	5.06 (0.88)	5.19 (0.85)	5.13 (1.00)
Tryglyceride (mmol/L) n = 1673	1.30 (0.45)	1.35 (0.51)	1.52 (0.61)	1.49 (0.49)
ApoA1 (g/L) n = 1626	1.59 (0.19)	1.60 (0.22)	1.57 (0.22)	1.56 (0.23)
ApoB (g/L) n = 1636	0.71 (0.15)	0.74 (0.17)	0.80 (0.16)	0.81 (0.23)
Ratio ApoB/ApoA1 n = 1636	0.45 (0.12)	0.47 (0.13)	0.51 (0.12)	0.53 (0.16)
FFA (mmol/L) n = 1559	0.29 (0.16)	0.30 (0.16)	0.35 (0.20)	0.40 (0.22)
***Child characteristics - at birth***				
Gestational age (days) n = 1727	279 (8.6)	281 (8.0)	282 (8.2)	282 (8.2)
Sex (%) n = 1727				
Boy	54.5	47.8	48.8	50.7
Girl	45.5	52.2	51.2	49.3
Birth weight (g) n = 1721	3307 (442)	3536 (482)	3608 (489)	3612 (531)
Std birth weight n = 1721	0.95 (0.11)	1.02 (0.13)	1.02 (0.13)	1.01 (0.13)
Breast feeding (%) n = 1719				
No breast feeding	7.6	14.0	16.0	25.3
< 1 month	10.6	5.4	9.7	8.0
1–3 months	24.2	27.5	23.7	24.0
>3 months	57.6	52.9	50.6	42.7
***Child characteristics - measurements at ages 5 years***				
Age (years) n = 1727	5.7 (0.5)	5.7 (0.5)	5.7 (0.5)	5.8 (0.5)
BMI (kg/m^2^) n = 1726	14.9 (1.3)	15.3 (1.3)	16.1 (1.5)	16.3 (2.1)
Overweight (%) n = 1726	1.5	6.1	16.5	24.0
Fat percentage (%) n = 1703	22.4 (5.6)	23.2 (5.9)	25.2 (6.7)	26.7 (6.80
WHtR n = 1725	0.45 (0.03)	0.45 (0.03)	0.46 (0.03)	0.46 (0.04)
Saturated fat intake (g/day) n = 1468	20.1 (5.7)	20.0 (5.7)	19.9 (7.0)	21.5 (7.9)
Screen time (hours/day) n = 1611	1.3 (0.7)	1.3 (0.9)	1.7 (1.1)	1.9 (1.2)

pBMI; pre pregnancy body mass index (kg/m^2^), WHtR; weight to height ratio, Std birth weight; standardized birth weight, TC; total cholesterol, TG; Triglyceride, FFA; free fatty acids.

* p<0.05.

** p<0.01.

*** p<0.001.

### Maternal pBMI and maternal lipids (a)

Table 2 reports the associations between pBMI and maternal lipid levels. pBMI was positively linearly associated with TC, TG, ApoB, ApoB/ApoA, FFA, and negatively linearly associated with ApoA1 levels (Model 1). These associations remained similar after adjustment for confounders (Model 2), with the exception of ApoA1 which was rendered non-significant.

**Table 2:** Association between pBMI and maternal lipids (n = 1727).

		pBMI	
		Model 1	Model 2
Maternal lipids^a^	Mean (SD)	β 95%CI	β 95%CI
TC (mmol/L)	5.08 (0.88)	0.02 (0.01; 0.03)	0.02 (0.01; 0.03)
TG (mmol/L)	1.38 (0.52)	0.02 (0.02; 0.03)	0.02 (0.01; 0.02)
ApoA1 (g/L)	1.59 (0.22)	−0.004 (−0.007; −0.0008)	−0.002 (−0.006; 0.007)
ApoB (g/L)	0.75 (0.17)	0.009 (0.007; 0.011)	0.008 (0.005; 0.01)
Ratio ApoB/ApoA	0.48 (0.13)	0.009 (0.007; 0.01)	0.006 (0.004; 0.009)
FFA (mmol/L)	0.31 (0.17)	0.008 (0.006; 0.01)	0.006 (0.004; 0.009)

pBMI; maternal pre-pregnancy BMI, TC; total cholesterol, TG; triglycerides, ApoA1; apolipoprotein A1, ApoB; apolipoprotein B, FFA; free fatty acids.

a standardized at median gestational age at blood sampling (13 weeks).

**Model 1**: Adjusted for gestational age at blood sampling.

**Model 2**: As model 1 with additional adjustment for age of mother, parity, ethnicity, height, years of education, alcohol, smoking, and hypertension.

### Maternal lipids and child adiposity (b)

Table 3 shows the associations between maternal lipids and childhood adiposity. Maternal FFA was positively linearly associated with the child's WHtR, fat percentage, BMI and risk for overweight (Table 3). These associations between maternal FFA and the child's fat percentage, BMI and risk for overweight remained significant after adjustment for confounders (model 2). Furthermore, maternal ApoB and TC were positively associated with the offspring's fat percentage, the effect size remained similar after adjustment for confounders. Finally, maternal TG was positively associated with their children's WHtR, also after adjustment for confounders. In the crude model TG was also positively associated with fat percentage and BMI; however, after adjustment for confounders the effect size dropped by 50% and these associations were no longer significant (Table 3).

**Table 3:** Association between maternal lipids and childhood adiposity (n = 1727).

	Child							
	WHtR * 100		Fat percentage (%)		BMI (kg/m^2^)		Risk for overweight	
	Model 1	Model 2	Model 1	Model 2	Model 1	Model 2	Model 1	Model 2
Maternal lipids	β (95% CI)	β (95% CI)	β (95% CI)	β (95% CI)	β (95% CI)	β (95% CI)	OR (95% CI)	OR (95% CI)
TC (mmol/L)	0.13 (−0.02; 0.28)	0.11 (−0.04; 0.26)	0.50 (0.18; 0.81)	0.47 (0.16; 0.79)	0.08 (−0.00; 0.15)	0.08 (−0.00; 0.15)	1.20 (1.00∶1.45)	1.17 (0.97; 1.42)
TG (mmol/L)	0.48 (0.23; 0.74)	0.31 (0.06; 0.57)	0.83 (0.30; 1.36)	0.36 (−0.17; 0.89)	0.19 (0.06; 0.32)	0.12 (−0.01; 0.25)	1.48 (1.10; 1.99)	1.24 (0.91; 1.68)
ApoA1 (g/L)	−0.01 (−0.63; 0.61)	0.02 (−0.60; 0.63)	0.31 (−0.96; 1.59)	0.89 (−0.37; 2.15)	−0.07 (−0.38; 0.24)	0.09 (−0.23; 0.40)	0.77 (0.34; 1.74)	1.14 (0.49; 2.63)
ApoB (g/L)	0.87 (0.09; 1.65)	0.57 (−0.21; 1.35)	3.19 (1.58; 4.79)	2.02 (0.44; 3.59)	0.45 (0.06; 0.84)	0.28 (−0.11; 0.67)	3.25 (1.25; 8.44)	2.00 (0.73; 5.49)
Ratio ApoB/ApoA	1.20 (0.14; 2.26)	0.80 (−0.26; 1.86)	3.64 (1.46; 5.83)	1.69 (−0.46; 3.85)	0.59 (0.06; 1.12)	0.23 (−0.30; 0.76)	4.85 (1.38; 17.07)	1.92 (0.50–7.42)
FFA (mmol/L)	1.09 (0.27; 1.91)	0.83 (−0.001; 1.65)	3.28 (1.58; 4.98)	2.02 (0.33; 3.71)	0.68 (0.27; 1.09)	0.45 (0.03; 0.86)	4.88 (1.94; 12.31)	3.06 (1.13; 8.30)

WHtR; weight to height ratio, TC; total cholesterol, TG; triglyceride, ApoA1; apolipoprotein A1, ApoB; apolipoprotein B, FFA; free fatty acids.

**Model 1**: Adjusted for gestational age at blood sampling, age of the child, sex.

**Model 2**: As model 1 with additional adjustment for age of mother, parity, ethnicity, height, years of education, alcohol, smoking, and hypertension. From the child: duration of exclusive breastfeeding, screen time hours/day and saturated fat intake.

In absolute numbers the increase in FFA from the 2.5^th^ percentile (0.11 mmol/L) to the 97.5^th^ percentile (0.77 mmol/L) would lead to an increase in offspring's fat percentage and BMI of 1.33% and 0.30 kg/m^2^, respectively. Furthermore, maternal FFA levels of 0.11 mmol/L (2.5^th^ percentile) led to an expected risk for overweight of 5.2% whereas FFA levels of 0.77 mmol/L (97.5^th^ percentile) increased this risk to 10.2%. The increase in TC and ApoB from the 2.5^th^ percentile (TC of 3.57 mmol/L and ApoB of 0.45 g/L) to the 97.5^th^ percentile (TC of 6.90 mmol/L and ApoB of 1.11 g/L) would lead to an increase of 1.56%. and 1.33% in fat percentage, respectively.

### Maternal pBMI and adiposity child (c)

A higher maternal pBMI was significantly associated with a greater adiposity of the child at age 5–6 years (Table 4). The effect size of the association between pBMI and adiposity of the child decreased only slightly after adjusting for the mothers' lipid profile; when a reduction was apparent, this was between 0% and 2%. When all lipids were added simultaneously to the regression model, the reduction of the effect size was somewhat higher (between 0% and 20%) and there was still a significant association between maternal pBMI and adiposity of the child. This indicates that, according to the causal-steps analyses, we found no proof that maternal lipid profile mediates the association between maternal pBMI and adiposity of the child. Also when mediation was assessed by testing the mediated effect computed as the product of regression coefficient estimates, we found no convincing proof for mediation effects of the maternal lipid profile in the association between maternal pBMI and adiposity of the child.

**Table 4:** **Association between maternal pBMI and childhood adiposity (n = 1727).**

	Child			
	WHtR * 100	Fat percentage	BMI (kg/m^2^)	Risk for overweight
	pBMI	pBMI	pBMI	pBMI
	β (95% CI)	β (95% CI)	β (95% CI)	OR (95% CI)
**Model 1**	0.15 (0.12; 0.19)	0.32 (0.24; 0.40)	0.11 (0.09; 0.13)	1.17 (1.13; 1.22)
**Model 2**	0.13 (0.09; 0.17)	0.21 (0.13; 0.29)	0.10 (0.08; 0.12)	1.15 (1.10; 1.20)
Model 2+TC (mmol/L)	0.13 (0.09; 0.17)	0.20 (0.11; 0.28)	0.10 (0.08; 0.12)	1.15 (1.10; 1.20)
Model 2+TG (mmol/L)	0.13 (0.09; 0.17)	0.20 (0.12; 0.28)	0.10 (0.08; 0.12)	1.15 (1.10; 1.20)
Model 2+ApoA1 (g/L)	0.13 (0.09; 0.17)	0.21 (0.13; 0.29)	0.10 (0.08; 0.12)	1.14 (1.09; 1.20)
Model 2+ApoB (g/L)	0.13 (0.09; 0.17)	0.20 (0.11; 0.28)	0.10 (0.08; 0.12)	1.14 (1.09; 1.19)
Model 2+Ratio ApoB/ApoA1	0.13 (0.09; 0.17)	0.20 (0.12; 0.28)	0.10 (0.08; 0.12)	1.14 (1.09; 1.19)
Model 2+FFA (mmol/L)	0.14 (0.09; 018)	0.19 (0.11; 0.27)	0.10 (0.08; 0.12)	1.14 (1.09; 1.19)
Model 2+ all lipids	0.12 (0.08; 0.16)	0.19 (0.10; 0.27)	0.08 (0.06; 0.11)	1.14 (1.09; 1.20)

pBMI; maternal pre-pregnancy BMI, WHtR; weight to height ratio, TC; total cholesterol, TG; triglycerides, ApoA1; apolipoprotein A1, ApoB; apolipoprotein B, FFA; free fatty acids.

**Model 1**: Adjusted for age and sex of the child.

**Model 2**: Adjusted for gestational age at blood sampling, age mother, parity, ethnicity, height, years of education, alcohol, smoking, and hypertension. From the child: age, sex, duration of exclusive breastfeeding, screen time hours/day and saturated fat intake.

In absolute numbers the increase in pBMI from the the 2.5^th^ percentile (18.2 kg/m^2^) to the 97.5^th^ percentile (32.1 kg/m^2^) would lead to increases of offspring's WHtR*100, fat percentage and BMI of 1.81, 2.91% and 1.39 kg/m^2^, respectively. Furthermore, a maternal pBMI of 18.2 kg/m^2^ (2.5^th^ percentile) led to an expected risk for overweight of 3.6% a pBMI of 32.1 kg/m^2^ (97.5^th^ percentile) increased this risk to 19.7%. Therefore, maternal pBMI appears to have a larger effect on the offspring's adiposity than the maternal lipid profile.

## Discussion

This study showed that overweight and obese mothers and mothers with pronounced FFA levels during early pregnancy were more likely to have children with overweight or obesity at age 5–6 years. The association between pBMI and the child's adiposity was largely independent of the maternal lipid profile during early pregnancy. Furthermore, a significant association was present between maternal FFA and offspring fat percentage, BMI and risk for overweight. Also maternal ApoB and TC were positively associated with the offspring's fat percentage and maternal TG was positively associated with their children's WHtR,

Our results are in line with others reporting a positive association between maternal pBMI and child adiposity, and between maternal pBMI and maternal lipid profile [6], [7], [28], [29]. To our knowledge no other studies have ever explored whether an association exist between maternal lipid profile and offspring adiposity. Our observations therefore add new evidence for the existence of foetal metabolic priming by intrauterine conditions, as in our case for maternal lipid profile. As both maternal lipid levels and pBMI were both independently associated with childhood's adiposity, and this implies a different (still unknown) mechanistic pathways.

In the present study maternal FFA levels during early pregnancy were related to offspring adiposity, whereas TG levels were only related to offspring WHtR. This was contrary to expectations as there is a strong biochemical link between FFA and TG levels; FFA are often derived from TG, also maternal TG reach the fetus as FFA as TG are lipolysed in the placenta to FFA. Adjustment for confounders strongly attenuated the association between TG and offspring adiposity. It seems that the confounders adjusted for in the present study were relatively important for this association.

Several mechanisms (or a combination of them) may underlie our finding that an adverse maternal lipid level programs their offspring for obesity, i.e. programming of the offspring's eating behavior, adipocyte development, post-natal influences, genetics and epigenetics. First, from animal studies we know that maternal high-fat diet during pregnancy can lead to structural hypothalamic changes [9] as well as to impaired leptin signalling in the arcuate nucleus of the hypothalamus and resistance to the anorectic actions of leptin [30], [31]. It is also known that fetal exposure to maternal FFA levels can induce endoplasmic reticulum stress and inflammation, with a subsequent increase in local cytokines in the hypothalamus [32]–[35]. The hypothalamus is involved in satiety and regulation of glucose and lipid metabolism and distribution of body fat. Therefore, changes in the hypothalamus may alter the eating behavior of the offspring towards an increased food intake and a preference for dietary fat [9]. Second, adverse maternal lipid levels may influence adipose tissue development [17], [36]. Early influences on adipocyte differentiation may have lifelong consequences on adiposity as the fat cell number remains stable throughout childhood. Third, the extrauterine environment, such as diet quality and parental behavior towards physical activity, is shared by mother and child and may contribute to the obesity risk [37], [38]. Fourth, research among twins shows that about 70% of the individual variation in adiposity between people is apparently due to genetic factors [39]. Although no genetic pathway has yet been established, common genes that are related to both lipid levels and adiposity might be involved. Finally, epigenetics might affect the association between maternal lipid levels during early pregnancy and their offspring's body composition. However, only limited evidence is available showing that epigenetic modifications are an important mediator between maternal obesity/maternal lipid status and long-term offspring outcomes [40]; further research is clearly required.

### Strengths and limitations

Strengths of this study are the large study population, the number of available covariables and the long follow-up period. Also, several childhood adiposity measures were available, i.e. besides BMI we also assessed the percentage of body fat and WHtR.

This study also has some limitations. First, blood collection for maternal plasma lipids was accomplished randomly i.e. on only one occasion and in a nonfasting state. The nonfasting state might have slightly diluted our results. However, in a cohort of initially healthy women, Bansal et al. found that nonfasting TG levels were associated with the prevalence of cardiovascular events; in that study fasting TG levels showed little independent relationship [41]. Also, another prospective cohort study found that elevated nonfasting TG levels were associated with increased risk of myocardial infarction, ischemic heart disease, and death [42]. As most of the day is spent in a nonfasting state, these nonfasting lipid levels may even more accurately estimate health risks than fasting lipid levels.

Second, selection bias may have occurred as this is a cohort study and selective loss to follow-up may have taken place. The women that participated in the ABCD biomarker study, but who were lost to follow-up during the health check measurement of their children at age 5 years(n = 1378), were significantly younger, less often of Dutch origin, had fewer years of education, and were less likely to consume alcohol (see Table S1). Both groups were comparable for maternal pBMI, standardized birth weight and gestational age. Due to this selective loss to follow-up and our exclusion criteria, the present results apply to a relatively healthy sample. Therefore, associations found in this study might be an underestimation of the effects of the whole community.

Third, although we adjusted for several factors we cannot exclude some residual confounding. Maternal pBMI was based on self-reported questionnaires. Self-reported height tends to be slighted overestimated and weight underestimated, resulting in a (possible) underestimation of BMI. Nevertheless, if present this underestimation will be apparent across almost the whole study population [43], [44]. We deliberately excluded birth weight as (according to our hypothesis) it lies within the causal pathway; maternal lipid status may influence offspring somatic growth [13] and thereby influence birth weight and finally influence childhood adiposity. Our explorative analyses showed that the results remained the same after the inclusion of birth weight (data not shown). Furthermore, gestational weight gain, specifically in combination with maternal pBMI, is associated with childhood adiposity [45]. Unfortunately information on gestational weight gain was not available, but we did have first trimester weight gain in a subsample (n = 1410). Explorative analysis with early gestational weight gain added to the full model attenuated our findings, but maternal FFA remained significantly associated with child's risk for overweight (data not shown). Moreover, gestation weight gain itself was positively associated with child BMI but not with child fat percentage and child WHtR. These results indicate that, besides maternal pBMI, excessive weight gain during gestation might also have the potential to program the child for adiposity; however, more research is needed to draw firm conclusions.

Finally, the model showing the risk of obesity might be an unstable one. This model includes many potential confounders, while there are few events. The results should be interpreted with caution.

### Implications

Higher maternal lipid levels during early pregnancy and maternal pBMI are associated with offspring adiposity at age 5–6 years. Because of the tracking of childhood adiposity into adulthood, we expect this increase in WHtR, fat percentage and child BMI to persist and become more pronounced later in life. Therefore, the impact of small increases at a young age might have detrimental effects on cardiovascular disease later in life. More research is needed to elucidate the etiology, and to develop preventive strategies to reduce adverse lipid profiles in the preconception phase to improve offspring's health.

### Conclusion

In conclusion, maternal pBMI and lipid profile during early pregnancy are independent factors related to childhood adiposity at young age.

## Supporting Information

Table S1 **Maternal and child characteristics of the study population and the group lost to follow-up.** pBMI; pre-pregnancy body mass index (kg/m^2^), TC; total cholesterol, TG; Triglyceride, * p<0.05, ** p<0.01, *** p<0.001.
(DOC)
