# Supplementary material for: Maternal Prepregancy BMI and Lipid Profile during Early Pregnancy Are Independently Associated with Offspring's Body Composition at Age 5–6 Years: The ABCD Study
Source: PLoS One. 2014 Apr 16;9(4):e94594. doi: 10.1371/journal.pone.0094594 (PMC3989215; doi:10.1371/journal.pone.0094594)
Supplement: Table S1 — Maternal and child characteristics of the study population and the group lost to follow-up. pBMI; pre-pregnancy body mass index (kg/m2), TC; total cholesterol, TG; Triglyceride, * p<0.05, ** p<0.01, *** p<0.001. (DOC) [file pone.0094594.s001.doc]

**Supplementary table.**

**Maternal and child characteristics of the study population and the group lost to follow-up.**

|  | **Study population** | **Lost to follow- up** |
| --- | --- | --- |
|  | **n=1728** | **n=1378** |
| ***Maternal characteristics*** | SD |  |
| Age (years) | 31.9 (4.3) | 30.1 (5.1)*** |
| pBMI (kg/m^2^) | 22.8 (3.6) | 22.9 (3.7) |
| Ethnicity (%) |  | *** |
| Dutch | 77.4 | 67.9 |
| Turkish | 2.1 | 4.2 |
| Moroccan | 4.1 | 6.5 |
| Other non-Western | 9.8 | 13.7 |
| Other Western | 6.7 | 7.6 |
| Primiparous (% yes) | 56.7 | 58.0 |
| Education after primary school (years) | 10.1 (3.5) | 8.7 (3.9)*** |
| Height (cm) | 169.7 (7.0) | 168.8 (7.1)*** |
| Hypertension (%) |  |  |
| Pre-existing | 2.4 | 3.2 |
| pregnancy induced | 8.5 | 9.1 |
| Smoking (yes, %) | 10.6 | 8.4* |
| Alcohol (yes, %) | 30.0 | 21.3*** |
| ***Lipids*** |  |  |
| Total cholesterol (mmol/L) | 5.12  0.93 | 5.14  0.94 |
| Tryglyceride (mmol/L) | 1.40  0.55 | 1.43  0.59 |
| ***Child characteristics*** |  |  |
| Gestational age (days) | 281 (8.0) | 281 (8.4) |
| Sex (%) |  |  |
| Boy | 48.4 | 48.8 |
| Girl | 51.6 | 51.2 |
| Birth weight (g) | 3542 (487) | 3513 (482) |
| Standardized birth weight | 1.01 (0.12) | 1.00 (0.12) |
| Breastfeeding (%) |  |  |
| No breastfeeding | 14.5 | 18.9*** |
| < 1 month | 6.3 | 9.2 |
| 1-3 months | 26.7 | 29.9 |
| >3 months | 52.4 | 42.0 |
